# Veratric Acid and the Cytotoxic Activity of *Handroanthus impetiginosus* Inner Bark Extract: No Association

**DOI:** 10.1002/cbdv.71721

**Published:** 2026-09-22

**Authors:** Gabriela Alberto Gil, Letícia Kakuda, Estéfani Maria Treviso, Mounika Aare, Lusânia Maria Greggi Antunes, Mandip Singh Sachdeva, Wanderley P. Oliveira

**Affiliations:** ^1^ School of Pharmaceutical Sciences of Ribeirão Preto University of São Paulo Ribeirão Preto Brazil; ^2^ College of Pharmacy and Pharmaceutical Sciences Florida A&M University Tallahassee USA

**Keywords:** cytotoxicity, *Handroanthus impetiginosus*, HPLC‐DAD, phytochemical marker, veratric acid

## Abstract

*Handroanthus impetiginosus* is a medicinal tree and a source of bioactive compounds whose cytotoxicity has often been associated with lapachol, α‐lapachone, and β‐lapachone. Because these compounds may occur at low concentrations, this study evaluated veratric acid as an alternative phytochemical marker of the cytotoxic activity of an inner bark extract. The hydroethanolic extract was obtained by dynamic maceration, lyophilized, and characterized for total polyphenol content, antioxidant activity, and phytochemical profile. Cytotoxic effects of the extract and veratric acid were assessed in HUVEC, MDA‐MB‐231, A549, and HepG2 cells. The extract reduced tumor‐cell viability more strongly than HUVEC viability under the tested conditions. In contrast, veratric acid at extract‐equivalent concentrations did not reduce cell viability. These findings indicate that veratric acid does not account for the cytotoxicity of the extract and is therefore unsuitable as a marker of this biological effect under the conditions evaluated.

## Graphical abstract

Physicochemical characterization, phytochemical profiling, and in vitro cytotoxicity assessment of *Handroanthus impetiginosus* inner bark extract show that veratric acid, despite being readily identified by HPLC‐DAD, does not account for the observed cytotoxicity. The findings indicate that other, still unidentified phytochemicals contribute to the extract's bioactivity and merit further investigation.

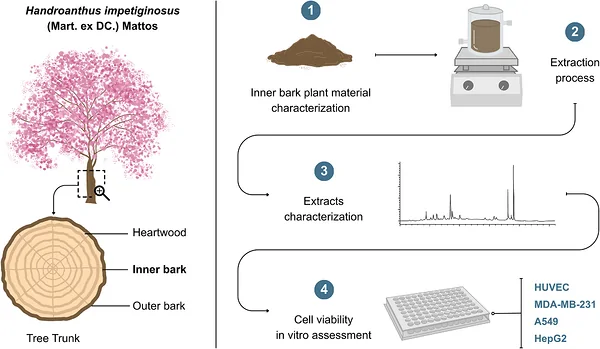

## 1 Introduction

Cancer remains a major global public health challenge, ranking among the leading causes of death worldwide and one of the greatest barriers to increasing life expectancy [1, 2]. According to Global Cancer Statistics, approximately 19.3 million new cancer cases and nearly 10 million cancer‐related deaths occurred worldwide in 2020 [2, 3]. World Health Organization (WHO) data also indicate that, in 2019, cancer was one of the four leading causes of death among individuals under 70 years of age in more than 100 countries [4]. Therefore, the development of novel and effective cancer therapies remains an urgent priority.

The search for novel cancer therapies has sustained interest in natural products, as many modern drugs originate from medicinal plants and traditional knowledge, including major anticancer agents such as vinblastine and paclitaxel [5]. Although this knowledge is increasingly at risk of being lost, WHO estimates that 70%–95% of the population in developing countries still relies on traditional medicine to meet their basic healthcare needs [6].

*Handroanthus impetiginosus* (Mart. ex DC.) Mattos (Bignoniaceae), also known as *Tabebuia avellanedae* Lorentz ex Griseb. and commonly referred to as *ipê‐roxo*, red lapacho, or taheebo, is a medicinal tree native to the tropical rainforests of Central and South America [7, 8]. Historical records indicate that its use dates back to the Inca civilization, and the species has long been employed in traditional medicine by native populations of these regions for the treatment of inflammation, infections, and cancer [9]. Owing to its extensive traditional use, phytochemical studies have identified the inner bark and heartwood as important sources of bioactive compounds, with reported anti‐inflammatory, antioxidant, antibacterial, antifungal, antiviral, and antineoplastic activities [10, 11, 12, 13, 14].

These biological activities have been associated with a diverse range of phytochemicals, particularly quinones, flavonoids, and phenolic acids [15]. Hamed et al. cataloged 163 compounds isolated from species of the genus *Tabebuia* spp., including iridoids (36), phenolic acids and derivatives (32), phenylpropanoids and phenylethanoids (7), lignans and neolignans (14), isocoumarins (7), flavonoids (14), quinones and naphthoquinones (50), sterols (2), and triterpenes (1) [16]. Despite this chemical diversity, the antineoplastic activity of *H. impetiginosus* has been primarily attributed to naphthoquinones such as lapachol, α‐lapachone, and β‐lapachone, which are therefore the most extensively investigated compounds in this species [7]. However, their relatively low concentrations in the plant hinder their extraction, identification, and quantification, thereby limiting further research [8, 17].

To further investigate this species, Jin et al. proposed veratric acid as an alternative phytochemical marker for *H. impetiginosus* [17]. This compound has been reported to exhibit antibacterial, antifungal, antioxidant, anti‐inflammatory, antihypertensive, and photoprotective properties [18, 19, 20, 21, 22]. However, evidence supporting an association between veratric acid and antineoplastic activity remains limited [23].

In this context, the present study evaluated the potential of veratric acid as a phytochemical marker of the cytotoxic activity of *H. impetiginosus*. To this end, the inner bark was characterized, and a hydroethanolic extract was obtained and evaluated for antioxidant activity, total polyphenol content, and phytochemical profile, with particular attention to veratric acid and the naphthoquinones lapachol, α‐lapachone, and β‐lapachone. The in vitro cytotoxic activity of the extract and veratric acid standard were also assessed in tumor and non‐tumor cell lines.

## 2 Results and Discussion

### 2.1 Characterization of Plant Material

The initial characterization focused on physicochemical attributes relevant to storage stability and extraction performance. The previously dried and ground inner bark exhibited low moisture content and a reduced amount of free water, suggesting favorable physicochemical characteristics for storage stability [24]. Moisture content and loss on drying values were 7.47 ± 0.24% (*w*/*w*) and 7.87 ± 0.11% (*w*/*w*), respectively, both being well below the recommended threshold of 14% for minimizing instability in plant materials [25]. In addition, the water activity value of 0.4617 ± 0.0100 was below the generally accepted maximum threshold of 0.5, indicating limited availability of free water and, consequently, a lower theoretical potential for water‐dependent deterioration and microbial growth [26].

Thermogravimetric analysis (TGA, Figure S1) and differential scanning calorimetry (DSC, Figure S2) were consistent with the low residual moisture content of the dried sample, as only about 10% mass loss was observed at temperatures close to 100°C, which is compatible with the evaporation of residual water [27].

Although solvent composition and the plant‐to‐solvent ratio are critical extraction parameters, particle size and morphology are also recognized as important factors influencing extraction efficiency. Mechanical size reduction applied during plant material pre‐processing increases the contact surface area between the solvent and the plant matrix and facilitates solvent diffusion into the solid matrix, thereby improving the extraction efficiency of polyphenol and other antioxidant constituents [28, 29, 30, 31]. Sieving analysis showed that the particle diameters corresponding to 10%, 50%, and 90% of the cumulative particle‐size distribution (D_10_, D_50_, and D_90_) were 99.93 ± 0.35 µm, 201.69 ± 2.39 µm, and 321.90 ± 1.42 µm, respectively. Based on these values, the SPAN index was 1.10 ± 0.01, suggesting a relatively narrow particle size distribution [32]. According to Baldelli and Aguilera (2025), particle sizes between 100 µm and 1 mm generally provide favorable extraction conditions by increasing the exposure of disrupted plant cells to the extraction solvent [31].

**FIGURE 1:**
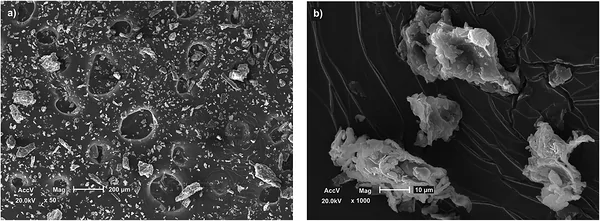
Scanning electron microscopy (SEM) images of the dried and ground inner bark of *Handroanthus impetiginosus*: (a) 50× magnification (scale bar: 200 µm) and (b) 1000× magnification (scale bar: 10 µm).

These findings were consistent with the scanning electron microscopy (SEM) photomicrographs (Figure 1a), which showed irregular, nonspherical particles, with a predominance of particles around or below 200 µm. At higher magnification (Figure 1b), SEM images revealed a rough and heterogeneous particle surface with irregularities resulting from the grinding process. These morphological characteristics are expected to favor solvent‐particle interactions by increasing the exposed surface area and facilitating solvent penetration into the plant matrix, which may contribute to improved extraction efficiency.

Due to the chemical complexity of plant materials, FTIR analysis is more suitable for identifying predominant functional groups than for the unequivocal assignment of individual constituents. According to Russo et al., plant matrices commonly show bands associated with lignocellulosic components, especially νO–H, aliphatic νC–H, and νC–O vibrations, corresponding here to bands I, II, and III, respectively [33]. Thus, band I (3600–3200 cm^−^^1^) can be attributed mainly to νO–H stretching of hydroxylated compounds, band II (around 2920–2850 cm^−^^1^) to aliphatic νC–H stretching, and band III (1800–1000 cm^−^^1^) to overlapping vibrations in the fingerprint region [33]. According to Szymczycha‐Madeja et al., this latter region is commonly associated with oxygenated constituents such as carboxylic acids, alcohols, and phenolic compounds, while the broad band in the 3200–3700 cm^−^^1^ range is characteristic of hydroxylated compounds [34].

**FIGURE 2:**
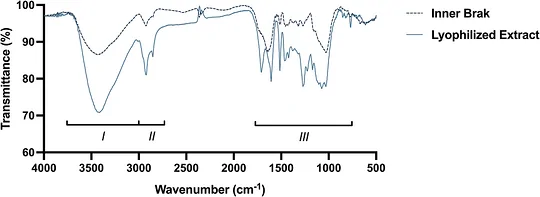
Fourier transform infrared (FTIR) spectra of the dried and ground inner bark of *Handroanthus impetiginosus* and the corresponding lyophilized extract, highlighting bands I, II, and III.

The maximum extractable matter (C_e_) in 70% (*v*/*v*) hydroethanolic solution was 0.52 ± 0.01% (*w*/*w*), indicating a low proportion of compounds soluble in the extraction solvent. The low C_e_ is consistent with the woody nature of the plant material, whose mass is largely composed of insoluble structural components such as cellulose and lignin [35]. This interpretation is also consistent with the FTIR profile, particularly bands I and III (Figure 2), which reflect the presence of hydroxylated and oxygenated constituents commonly found in lignocellulosic plant matrices [33].

### 2.2 Characterization of Liquid and Lyophilized Extract

A hydroethanolic extract was obtained by dynamic maceration with heating, followed by vacuum filtration. The resulting extract solution showed a solid content (*C*_s_) of 0.37 ± 0.03% (*w*/*w*). Although this value was low, the extraction efficiency (*η*), calculated as the ratio between *C*_s_ and *C*_e_, reached 71.2%, indicating effective recovery of the solvent‐extractable fraction.

**FIGURE 3:**
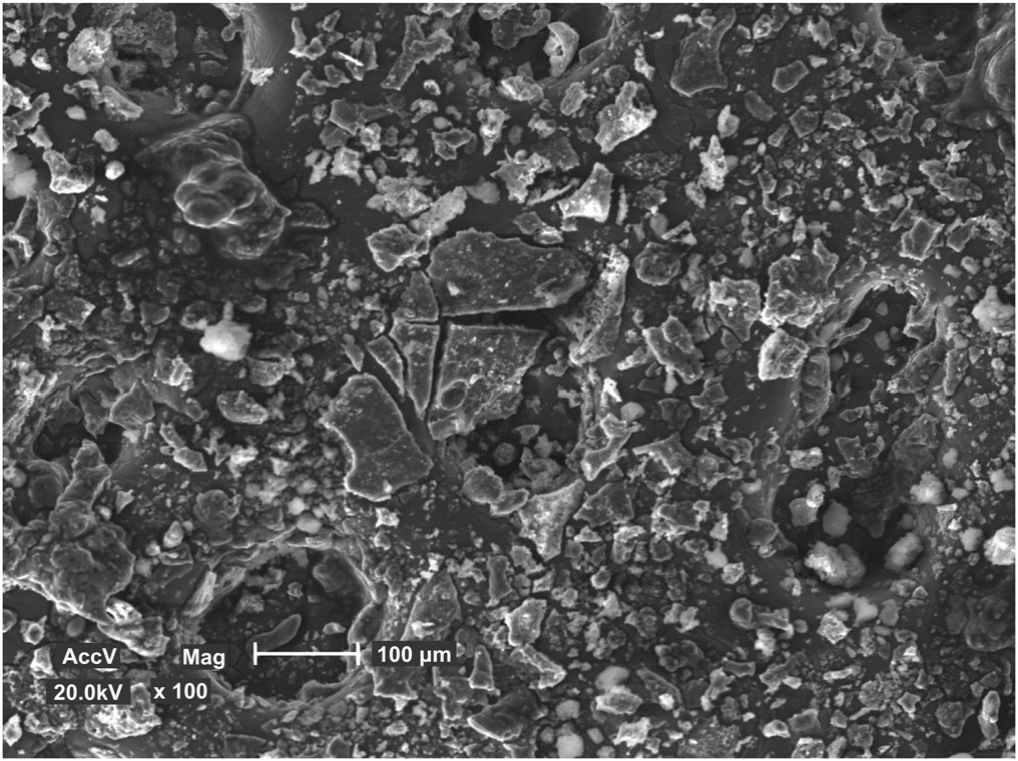
Scanning electron microscopy (SEM) image of the lyophilized inner bark extract at 100× magnification (scale bar: 100 µm).

The extract solution was concentrated by rotary evaporation to remove ethanol and subsequently lyophilized, yielding a free‐flowing powder that could be readily reconstituted in the original hydroethanolic solvent [36]. SEM analysis of the lyophilized extract (Figure 3) revealed predominantly irregular and non‐spherical particles, many of them with dimensions below approximately 100 µm. The absence of large compact agglomerates and the predominance of small irregular particles are consistent with the free‐flowing behavior and good redispersion of the lyophilized extract observed during sample preparation.

FTIR analysis of the extract showed a profile generally similar to that of the plant material, with more intense bands in regions I and III (Figure 2). The increased intensity of these bands is consistent with enrichment in soluble oxygenated constituents after extraction, especially compounds containing hydroxylated and other oxygenated functional groups, such as phenolic acids, alcohols, sugars, and related polar constituents [34].

This interpretation is consistent with the total polyphenol content and antioxidant activity of the extract. Based on the pyrogallol equivalent (PE) calibration curve (Figure S3), the extract showed a total polyphenol content of 32.95 ± 4.46 µg_PE_/mg. In addition, a relatively low extract concentration was required to reduce 50% of the initial DPPH radical content (IC_50‐DPPH_ = 33.90 µg/mL, Figure S4), indicating measurable DPPH radical‐scavenging activity. This finding agrees with the well‐established relationship between polyphenol content and antioxidant capacity, since phenolic compounds are effective radical scavengers [31, 37, 40]. Consistently, Ganesan et al. evaluated fifteen medicinal plant species and reported a positive correlation between total polyphenol content and in vitro antineoplastic activity [39]. This association has been attributed, at least in part, to the involvement of oxidative stress in tumor development and progression, making antioxidant phytochemicals attractive candidates for anticancer drug discovery [39]. Nevertheless, these results should be interpreted with caution, as the antioxidant capacity of the extract was assessed using only a single in vitro assay. Additional complementary assays would provide a more comprehensive evaluation of its antioxidant properties.

**FIGURE 4:**
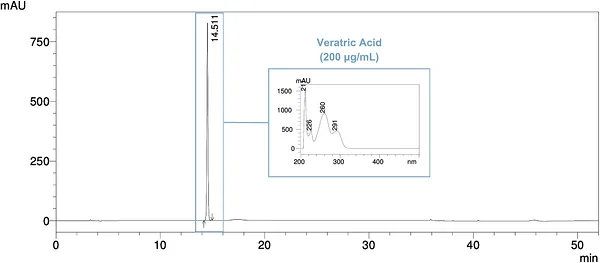
HPLC‐DAD chromatogram of the veratric acid standard solution (200 µg/mL) recorded at 254 nm. The inset shows the DAD spectrum of the peak at retention time of 14.511 min, with absorption maxima at approximately 211, 226, 260, and 291 nm.

**FIGURE 5:**
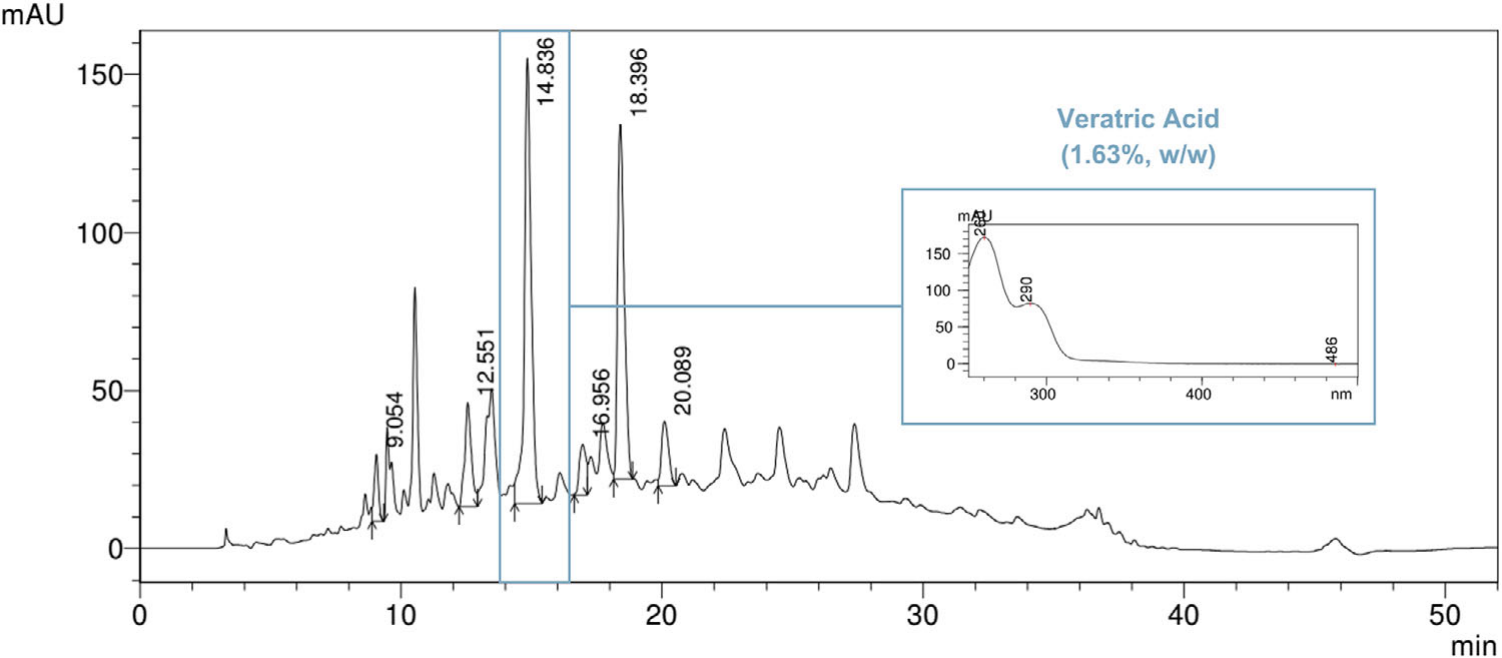
HPLC‐DAD chromatogram of the lyophilized inner bark extract (5000 µg/mL) recorded at 254 nm. The highlighted peak at retention time of 14.836 min was assigned to veratric acid by comparison of retention time and DAD spectrum with the reference standard. The inset shows the corresponding DAD spectrum, with absorption maxima at approximately 260 and 290 nm.

High‐performance liquid chromatography with diode array detector (HPLC‐DAD) analysis was performed to characterize the phytochemical profile of the extract, with particular attention to veratric acid (Figure 4) and the naphthoquinones lapachol (Figure S5), α‐lapachone (Figure S6), and β‐lapachone (Figure S7). Under the analytical conditions employed, lapachol, α‐lapachone, and β‐lapachone were not detected in the extract, in agreement with previous reports indicating the low abundance of these compounds in *H. impetiginosus* [8, 17]. In contrast, veratric acid was detected, and its content was estimated at 1.63% (*w*/*w*), as shown in Figure 5, supporting its use as a readily detectable analytical constituent of this extract.

The observed phytochemical profile can be partially explained by the extraction conditions employed. Solvent polarity is one of the main factors governing the extraction efficiency of plant metabolites, with hydroorganic mixtures generally providing high extraction yields for phenolic compounds [40]. Veratric acid is a low‐molecular‐weight phenolic acid whose extraction is favored by polar protic solvents, including methanol and ethanol [41]. Accordingly, the 70% (*v*/*v*) hydroethanolic solvent selected in the present study provides a hydroorganic extraction system suitable for recovering polar phenolic constituents, particularly when associated with heating [40, 42].

In contrast, the extraction of naphthoquinones is frequently favored by aprotic organic solvents, such as acetone and acetonitrile, depending on the extraction conditions and the target compound [43]. Therefore, the extraction conditions adopted in the present study may have preferentially enriched phenolic constituents, contributing to the absence of detectable levels of lapachol, α‐lapachone, and β‐lapachone. However, the lack of detection should not be interpreted as evidence of their absence in the plant material, since these compounds may have been present at concentrations below the detection limit of the analytical method. Indeed, previous studies using ethanol‐based extraction procedures have reported the presence of these naphthoquinones in *H. impetiginosus*, although generally at relatively low concentrations [44, 45, 46].

In addition to extraction conditions, abiotic, and biological factors may also contribute to variations in the phytochemical composition of medicinal plants [47, 48, 49]. Therefore, differences among studies may reflect not only methodological aspects but also the intrinsic chemical variability of the plant material. Although the influence of these factors on secondary metabolite production is well established in phytochemistry studies, their effects on the phytochemical profile of *H. impetiginosus* have been only sparsely investigated. Consequently, the phytochemical profile observed in the present study likely reflects the combined influence of the extraction conditions employed and the intrinsic chemical variability of the plant material.

### 2.3 Cell Viability Assessment

Cell viability assays are widely used to assess the cytotoxic effects of compounds in vitro [50]. In the present study, cell viability was determined by the resazurin assay for lyophilized extract (E) and veratric acid standard (VA) in HUVEC, MDA‐MB‐231, A549 and HepG2 cells.

**FIGURE 6:**
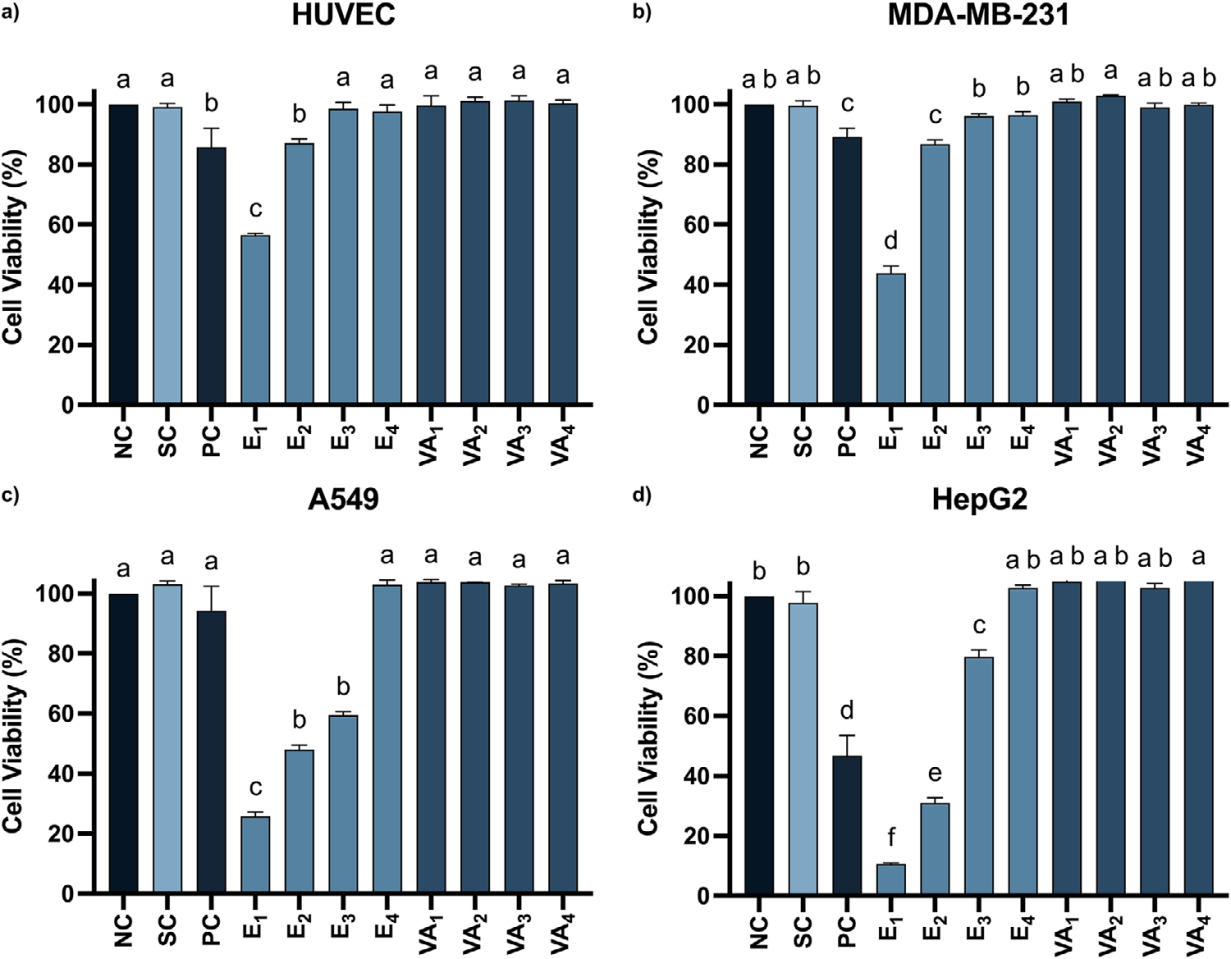
Cell viability (%) of HUVEC, MDA‐MB‐231, A549, and HepG2 cells after exposure to *Handroanthus impetiginosus* inner bark extract (*E*_1_–*E*_4_), veratric acid standard (VA_1_–VA_4_), solvent control (SC), negative control (NC), and positive control (PC). *E*_1_–*E*_4_ correspond to extract concentrations of 500.0, 250.0, 125.0, and 62.5 µg/mL, respectively, whereas VA_1_–VA_4_ correspond to veratric acid concentrations of 8.15, 4.07, 2.04, and 1.02 µg/mL, respectively. SC contained 1.75% (*v*/*v*) ethanol, and PC contained 4% (*v*/*v*) DMSO. Within each panel, bars sharing the same letter are not significantly different, whereas bars with different letters differ significantly according to one‐way ANOVA followed by Tukey's multiple comparisons test (*p* < 0.05). The corresponding mean differences and adjusted *p*‐values are provided in Table S1. Error bars represent the standard deviation (SD). Data represent mean ± SD of three technical replicates per treatment (*n* = 3).

The extract exhibited a concentration‐dependent cytotoxic effect against the three tumor cell lines investigated, as shown in Figure 6. Statistically significant reductions in cell viability compared with the negative control (NC) were observed at 500, 250, and 125 µg/mL for A549 cells (adjusted *p* < 0.0001), at 500, 250, and 125 µg/mL for HepG2 cells (adjusted *p* < 0.0001), and at 500 and 250 µg/mL for MDA‐MB‐231 cells (adjusted *p* < 0.0001) (Table S1). Consistent with these findings, A549 and HepG2 were the most sensitive cell lines, presenting the lowest IC_50_ values (179.44 ± 8.68 and 185.10 ± 3.77 µg/mL, respectively), whereas MDA‐MB‐231 showed lower susceptibility to the extract. These findings are consistent with previous reports describing cytotoxic effects of *H. impetiginosus* extracts and fractions against MDA‐MB‐231, A549, and HepG2 cells [11, 38].

As shown in Figure 6, the solvent control (SC) did not significantly affect cell viability compared with the NC in any of the evaluated cell lines (Table S1), indicating that the effects observed for the extract (*E*_1_–*E*_4_) were attributable to its constituents rather than to the ethanol used for solubilization. This result is consistent with previous studies demonstrating that ethanol concentrations equal to or lower than 2.5% (*v*/*v*) do not significantly affect the viability of cell lines such as MDA‐MB‐231 and HepG2 [51].

In the non‐tumor HUVEC cell line, the extract showed lower cytotoxicity than in the tumor cell lines, as cell viability remained above 50% even at the highest tested concentration (500 µg/mL). This finding suggests some degree of selectivity under the conditions evaluated. Similarly, Nahar et al. reported that a 60% hydroethanolic extract prepared from roasted *H. impetiginosus* plant material exhibited significant cytotoxic activity against A549 cells at 500 µg/mL while not significantly affecting the viability of the non‐tumor human keratinocyte cell line (HaCaT) at the same concentration, showing a similar pattern of greater cytotoxicity toward tumor than non‐tumor cells [38]. However, this interpretation should be made with caution, as it is based on a single non‐tumor cell line and one in vitro viability assay.

No tested concentration of veratric acid significantly reduced cell viability relative to the NC (Table S1). Furthermore, direct comparison between the highest extract concentration (*E*_1_) and the equivalent concentration of veratric acid (VA_1_) revealed significant differences in the tumor cell lines (Table S1), reinforcing that veratric acid alone does not account for the cytotoxic activity observed for the *H. impetiginosus* extract. This interpretation is supported by the literature, since cytotoxic effects of isolated veratric acid have only been reported at higher concentrations than those evaluated in the present study, including IC_50_ values of 41.2 µg/mL for HepG2 and 13.6 µg/mL for A549 [23]. Likewise, Wang et al. did not observe cytotoxicity of veratric acid alone at concentrations up to 20.0 µM (approximately 3.64 µg/mL), including in A549 cells [52].

The lack of cytotoxicity of veratric acid at extract‐equivalent concentrations suggests that other metabolites contribute to the biological activity of the extract. Although the antineoplastic activity of *H. impetiginosus* is often associated with lapachol and related naphthoquinones, these compounds were not detected under the analytical conditions employed in the present study [53, 54]. Their absence may indicate concentrations below the detection limit of the analytical method or the contribution of other, less explored constituents, including additional naphthoquinone derivatives such as dehydro‐α‐lapachone, whose cytotoxic potential has also been demonstrated in *H. impetiginosus* [55, 56]. Moreover, the phytochemical composition of *H. impetiginosus* is highly complex, with numerous classes of constituents reported in the literature. This chemical diversity makes it difficult to attribute the observed cytotoxic activity to a single constituent [16, 57]. Previous studies have also reported a positive correlation between total polyphenol content and in vitro antineoplastic activity [39]. Considering the measurable total polyphenol content of the extract determined in the present study, it is plausible that phenolic constituents, acting individually or synergistically, also contribute to the observed cytotoxic effect.

Overall, the results demonstrate that although veratric acid is readily detectable in the extract by HPLC‐DAD, it does not account for the cytotoxic activity observed under the evaluated conditions. Future studies employing bioassay‐guided fractionation and complementary phytochemical analyses may help identify the constituents responsible for the observed cytotoxicity and clarify whether synergistic interactions among metabolites contribute to the biological activity of the extract.

## 3 Conclusions

The hydroethanolic extract obtained from the inner bark of *H. impetiginosus* reduced the viability of the three tumor cell lines evaluated in vitro while showing a smaller effect on HUVEC cells under the tested conditions. This differential response warrants further investigation but should not be interpreted as evidence of therapeutic selectivity. Chemical characterization showed that veratric acid was detectable in the extract, whereas lapachol, α‐lapachone, and β‐lapachone were not detected under the analytical conditions employed. Veratric acid, at extract‐equivalent concentrations, did not reduce cell viability and therefore does not account for the cytotoxic activity of the extract under the conditions evaluated. Other constituents, potentially acting individually or synergistically, may contribute to the observed activity. Bioassay‐guided fractionation and broader phytochemical profiling are needed to identify the compounds associated with these effects.

## 4 Experimental Section

### 4.1 Characterization of Plant Material

The inner bark, commercially sold as *H. impetiginosus*, was purchased in March 2023 from Casa das Ervas Medicinais, a supplier located in the district of Palmares, Parintins, Amazonas, Brazil (2°38'05''S, 56°44'07''W). Access to the genetic material was registered in SisGen, the Brazilian national system for the management of genetic heritage and associated traditional knowledge, under registration number ACAB58C. The material was used according to the supplier's identification. Because it was commercially acquired, plant age, exact collection site, harvest date/season, and a voucher specimen were not available for this study.

The plant material was dried in a forced‐air oven (Nova Ética, model 420CLD‐300, Brazil) at 40°C until constant weight and then ground in a knife mill (Marconi, model MA 680, Brazil) equipped with a 20‐mesh sieve (833 µm) [58]. The dried and ground material was characterized for residual moisture content by Karl Fischer titration (Metrohm, model 870 KF Titrino Plus, Switzerland); loss on drying at 105°C by gravimetric analysis using a moisture analyzer (Sartorius Stedim Biotech GmbH, model MA 35, Göttingen, Germany); water activity at 25°C (AquaLab 4TEV, Decagon Devices, USA); and maximum extractable matter (*C*_e_) in 70% (*v*/*v*) hydroethanolic solution [36, 58, 59].

Particle size distribution was determined by mechanical sieving using a Tyler series of sieves (600 to 75 µm). From the cumulative particle‐size distribution, the diameters corresponding to 10%, 50%, and 90% of the sample (*D*_10_, *D*_50_, and *D*_90_, respectively) were obtained and used to calculate the SPAN index according to Equation 1.

(1) $$\mathrm{SPAN}=\frac{D_{90}-D_{10}}{D_{50}}$$

The plant material was further characterized by scanning electron microscopy (SEM, Zeiss EVO 50) to obtain micrographs at 50× and 1000× magnification; Fourier transform infrared spectroscopy (FTIR, Prestige‐21, Shimadzu); thermogravimetric analysis (TGA, Perkin Elmer, model TGA 4000, U.S.A.) from 30°C to 900°C at a heating rate of 20°C/min, at an N2 flow rate of 20 mL/min; and differential scanning calorimetry (DSC, Perkin Elmer, model Jade DSC, U.S.A.) from 20°C to 300°C at a heating rate of 10°C/min, at an N2 flow rate of 30 mL/min. TGA and DSC data were analyzed using Pyris software (Perkin Elmer, U.S.A.).

### 4.2 Extraction, Concentration, and Lyophilization

The extract was prepared by dynamic maceration in a jacketed vessel with temperature control provided by an ultrathermostatic bath (Marconi, model MA‐184, Brazil). The extraction was performed using a 70% (*v*/*v*) hydroethanolic solution at 70°C for 120 min, with a plant material‐to‐solvent ratio of 1:20 (*w*/*v*). The resulting solutions were vacuum‐filtered and concentrated using a rotary evaporator to remove ethanol. Prior to lyophilization, colloidal silicon dioxide (Aerosil 200, Evonik, Germany) was added to the concentrated extracts as a drying aid at 5% (*w*/*w*, dry basis). The mixtures were frozen at −20°C for 24 h, followed by 4 h at −80°C, and subsequently lyophilized for 72 h in a freeze‐dryer (Thermo Fisher Scientific, model SNL108B, USA) [60].

### 4.3 Characterization of Liquid and Lyophilized Extract

The extract solution was characterized for solid content (*C*_s_) according to the method described by Souza, Bott, and Oliveira (2007), adapted for use with a moisture analyzer [36]. Extraction efficiency (*η*) was calculated as the ratio between *C*_s_ and *C*_e_. The *C*_e_ value was determined at a 1% (*w*/*w*) plant‐to‐solvent ratio and mathematically adjusted to the 5% (*w*/*w*) ratio used for extract preparation.

The lyophilized extract was evaluated for total polyphenol content and antioxidant activity using a UV–vis spectrophotometer (HP 8453, USA). Total polyphenol content was determined by the Folin–Denis method using pyrogallol (Orgânica, Brazil) as the reference standard [61]. The lyophilized extract was redispersed in Milli‐Q water at 1.0 mg/mL, homogenized in an ultrasonic bath for 5 min, and filtered. The resulting solution was diluted 1:5 (*v*/*v*), and a 500 µL aliquot was transferred to a test tube containing 500 µL of Folin–Denis reagent and 4.0 mL of saturated sodium carbonate (Na_2_CO_3_) solution. After vortex mixing, the reaction was allowed to proceed for 2 min, and the absorbance was measured at 750 nm against a Milli‐Q water blank. A pyrogallol stock solution (500 µg/mL) was used to prepare a seven‐point calibration curve (5–35 µg/mL) following the same analytical procedure applied to the samples. Sample and standard solutions were analyzed in three technical replicates, and the results were expressed as µg pyrogallol equivalents per mg of lyophilized extract (µg_PE_/mg).

Antioxidant activity was determined using the 2,2‐diphenyl‐1‐picrylhydrazyl (DPPH) radical scavenging assay (Sigma–Aldrich, USA) [62]. The lyophilized extract was redispersed in absolute ethanol at 1.5 mg/mL and sonicated for 5 min to ensure complete solubilization. Aliquots of the stock solution were mixed with 0.1 M acetate buffer (pH 5.5), adjusting the ethanol volume to obtain eight extract concentrations (0.006–0.090 mg/mL) while maintaining a constant final reaction volume before DPPH addition. The stock solution was prepared by dispersing 37.5 mg of lyophilized extract in 25 mL of absolute ethanol and sonicated for 5 min before use. The reaction was initiated by adding 500 µL of a 250 µM DPPH solution to each tube, followed by vortex mixing and incubation for 20 min at room temperature. Absorbance was measured at 517 nm. The DPPH control consisted of 1000 µL of acetate buffer, 1000 µL of absolute ethanol, and 500 µL of DPPH solution, whereas the blank consisted of 1000 µL of acetate buffer and 1500 µL of absolute ethanol. All concentrations were analyzed in three technical replicates. The concentration‐response relationship was analyzed by nonlinear regression using a four‐parameter logistic model (log[inhibitor] vs. response, variable slope), in which the extract concentrations were log_10_‐transformed prior to curve fitting. Analyses were performed in GraphPad Prism version 10.2.1 (GraphPad Software, USA). Antioxidant activity was expressed as the half‐maximal inhibitory concentration (IC_50‐DPPH_), defined as the extract concentration required to inhibit 50% of the initial DPPH radicals. The lyophilized extract was also characterized by FTIR and SEM, the latter providing micrographs at 100× magnification.

For chromatographic analysis, the lyophilized extract was redispersed in methanol at 5000 µg/mL. The high‐performance liquid chromatography with diode array detection (HPLC‐DAD) method was adapted from previous studies [17, 63, 64] to obtain the chromatographic profile of the extract and to assess the presence of veratric acid (Sigma–Aldrich, Germany), lapachol (Sigma–Aldrich, USA), α‐lapachone (Ambeed, USA), and β‐lapachone (Ambeed, USA) by comparison with pure standards. A methanolic standard solution containing 200 µg/mL of each compound was prepared for comparison of retention times and UV spectra. All samples were homogenized in an ultrasonic bath for 5 min, filtered through a 0.45 µm membrane filter, and transferred to capped vials prior to injection. Analyses were performed on an HPLC system (Shimadzu CBM‐20, Japan) equipped with a photodiode array detector (SPD‐M20A), pumps (LC‐6AD), an autosampler (SIL‐10AF), and LC Solution software (Version 1.24 SP1). Separation was achieved on a C18 column (Shimadzu Shim‐pack CLC‐M, 250 mm × 4.6 mm i.d., 5 µm). The mobile phase consisted of acidified water containing 1% (*v*/*v*) acetic acid (A) and methanol (B). Gradient elution from 10% to 100% B over 52 min was carried out at a flow rate of 1.0 mL/min, with the column maintained at 30°C. The injection volume was 10 µL, and detection was performed at 254 nm. The identity of veratric acid and the investigated naphthoquinones in the lyophilized extract was established by comparison of retention times and DAD spectra with those of the corresponding reference standards. The veratric acid content in the extract was estimated by external standard comparison using the ratio between the chromatographic peak area of the sample and that of the veratric acid standard, assuming a linear detector response within the concentration range employed.

### 4.4 Cell Viability Assessment

Human umbilical vein endothelial cells (HUVEC, CRL‐1730, RRID:CVCL_2959), human hepatocarcinoma cells (HepG2, HB‐8065, RRID:CVCL_0027), human triple‐negative breast adenocarcinoma cells (MDA‐MB‐231, HTB‐26, RRID:CVCL_0062), and human lung carcinoma cells (A549, CCL‐185, RRID:CVCL_0023) were obtained from the American Type Culture Collection (ATCC).

HUVEC and HepG2 cells were cultured in Dulbecco's Modified Eagle Medium (DMEM, Gibco), pH 7.4, supplemented with 100× penicillin‐streptomycin, 3.7 g/L sodium bicarbonate, and 10% fetal bovine serum (FBS). MDA‐MB‐231 and A549 cells were cultured in Roswell Park Memorial Institute medium (RPMI, Gibco) supplemented with the same components. Cells were maintained in an incubator at 37°C with 5% CO_2_ and 95% humidity. All experiments were conducted using cells between passages 3 and 8 post‐thaw. All cell culture procedures were performed under sterile conditions.

Lyophilized extracts and veratric acid solutions were prepared immediately before treatment as follows. Lyophilized extract was redispersed in 70% (*v*/*v*) hydroethanolic solution and homogenized in an ultrasonic bath for 5 min to obtain a stock solution of 200 000 µg/mL. Working concentrations ranging from 500.0 to 62.5 µg/mL (*E*_1_–*E*_4_) were prepared by serial dilution in the appropriate culture medium. For comparison, veratric acid (VA) was prepared in the same way, resulting in a stock solution of 326 µg/mL, which was serially diluted in culture medium to obtain working concentrations ranging from 8.15 to 1.02 µg/mL (VA_1_–VA_4_). The concentration of VA_1_ corresponded to the concentration of veratric acid present in *E*_1_. To account for possible solvent effects, a SC was prepared by diluting 125 µL of the 70% (*v*/*v*) hydroethanolic solution with culture medium to a final volume of 5 mL, resulting in a final ethanol concentration of 1.75% (*v*/*v*), corresponding to the ethanol concentration in *E*_1_ and VA_1_. Dimethyl sulfoxide (DMSO, 4% *v*/*v*) was used as the positive control (PC), and fresh culture medium was used as the NC.

**FIGURE 7:**
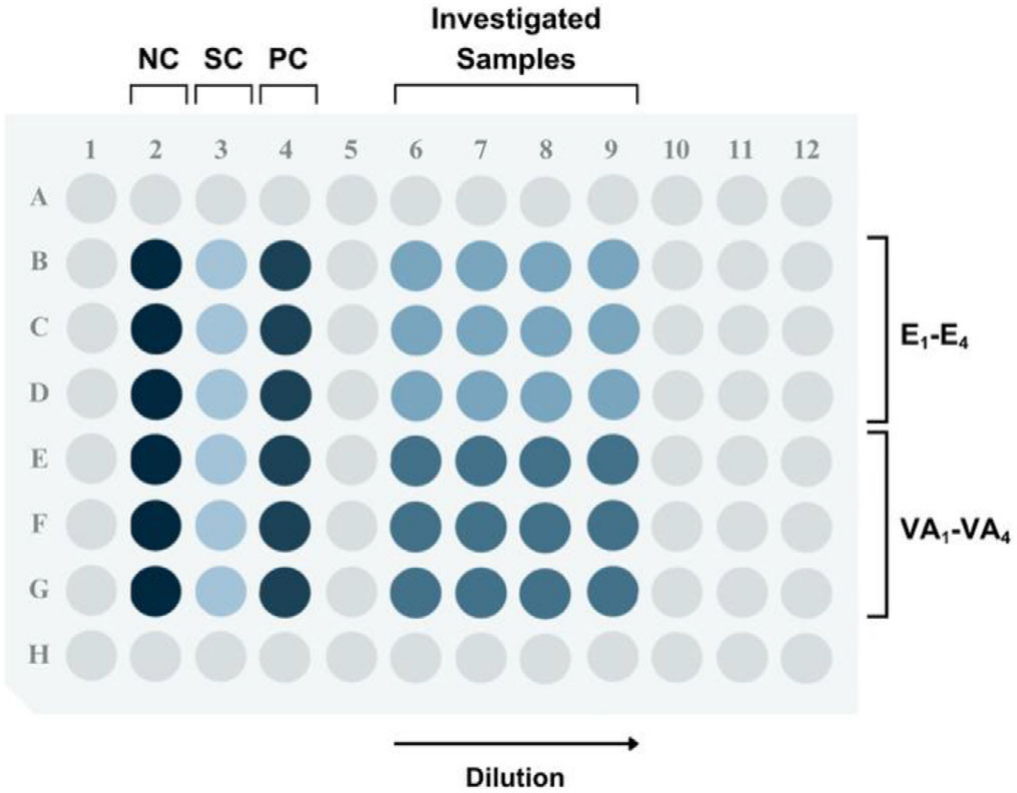
Illustration of the sample arrangement in the 96‐well plate. The assay included four concentrations of *Handroanthus impetiginosus* extract (*E*_1_–*E*_4_, 500.0–62.5 µg/mL), four concentrations of veratric acid standard (VA_1_–VA_4_, 8.15–1.02 µg/mL), one solvent control (SC, 1.75% *v*/*v* ethanol), one negative control (NC, fresh culture medium), and one positive control (PC, 4% *v*/*v* DMSO). Each sample was evaluated in three technical replicates.

For cytotoxicity evaluation, cells were seeded in 96‐well plates at a density of approximately 1 × 10^4^ cells/well and allowed to adhere for 48 h in the incubator. After the 48 h adhesion period, cells were treated with increasing concentrations of the extract (*E*_1_–*E*_4_), veratric acid (VA_1_‐VA_4_), SC, PC, or NC for 24 h. All treatments were plated in three technical replicates in 96‐well plates according to the layout shown in Figure 7.

Cell viability was assessed by the resazurin assay following the protocol described by Page, Page, and Noel (1993) [65]. Briefly, 15 µL of a reagent solution containing 398 µM resazurin (AlamarBlue; Sigma–Aldrich, USA) in phosphate‐buffered saline (PBS, pH 7.4) was added to each well, and the final volume was adjusted with 85 µL of culture medium. After incubation for 4 h at 37°C protected from light, fluorescence was measured using excitation and emission filters of 530 and 590 nm, respectively, in a Synergy 2 fluorescence spectrometer (Biotek, Winooski, USA).

Cell viability data were normalized to the NC (defined as 100% viability) and expressed as percentage before statistical analysis. For each cell line, differences among treatments were analyzed by one‐way ANOVA followed by Tukey's multiple comparisons test (two‐sided; *α* = 0.05). Adjusted *p*‐values were calculated for all pairwise comparisons. Half‐maximal inhibitory concentration (IC_50_) values were estimated by linear interpolation between the two concentrations immediately surrounding 50% cell viability. Data are presented as mean ± standard deviation (SD). Statistical analyses were performed using GraphPad Prism Version 10.2.1 (GraphPad Software, USA).

### 4.5 Statistical Analysis

Data preprocessing, statistical analyses, and curve fitting were performed according to the requirements of each experimental method, as described in the corresponding sections of the Experimental Section. Unless otherwise stated, data are presented as mean ± standard deviation (SD). Statistical analyses were performed using GraphPad Prism Version 10.2.1 (GraphPad Software, USA), adopting a significance level of *α* = 0.05. Method‐specific information regarding data normalization, sample size, statistical tests, post hoc analyses, and model fitting is provided in the respective experimental procedures.

## Author Contributions

Conceptualization: G.A.G., L.K., and W.P.O. Methodology: G.A.G., L.K., E.M.T., M.A., L.M.G.A., M.S.S., and W.P.O. Software: G.A.G. Validation: G.A.G., L.K., and W.P.O. Formal analysis: G.A.G., E.M.T. Investigation: G.A.G. Resources: L.M.G.A., M.S.S., and W.P.O. Data curation: G.A.G., L.K., and E.M.T. Roles/writing – original draft: G.A.G. Writing – review and editing: L.K., E.M.T., M.A., L.M.G.A., M.S.S., and W.P.O. Visualization: G.A.G., L.K., and W.P.O. Supervision: L.M.G.A., M.S.S., and W.P.O. Project administration: W.P.O. Funding acquisition: L.M.G.A., M.S.S., and W.P.O. All authors have read and agreed to the published version of the manuscript .

## Conflicts of Interest

The authors declare no conflicts of interest.

## Supporting information




**Supporting File 1**: cbdv71721‐sup‐0001‐SuppMat.docx.

## Data Availability

The data presented in this study are available on request from the corresponding authors.
