# Supplementary material for: Veratric Acid and the Cytotoxic Activity of Handroanthus impetiginosus Inner Bark Extract: No Association
Source: Chem Biodivers. 2026 Sep 22;23(9):e71721. doi: 10.1002/cbdv.71721 (PMC13598351; doi:10.1002/cbdv.71721)
Supplement: Supplementary file 1 [file CBDV-23-e71721-s001.docx]

**Veratric Acid and the Cytotoxic Activity of *Handroanthus impetiginosus* Inner Bark Extract: No Association**

Gabriela Alberto Gil^a,b^, Letícia Kakuda^a^, Estéfani Maria Treviso^a^, Mounika Aare^b^, Lusânia Maria Greggi Antunes^a^, Mandip Singh Sachdeva^b^, and Wanderley P. Oliveira^a,^*

^a^ School of Pharmaceutical Sciences of Ribeirão Preto, University of São Paulo, Ribeirão Preto 14040-903, Brazil

^b^ College of Pharmacy and Pharmaceutical Sciences, Florida A&M University, Tallahassee, U.S.A

(*Corresponding Author: [wpoliv@usp.br](mailto:wpoliv@usp.br))

Supplementary Material

**Figure SM1.** Thermogravimetric Analysis (TGA) curve of the dried and ground inner bark of *Handroanthus impetiginosus*, recorded from 30 to 900 ºC at 20 ºC/min under N_2_ (20 mL/min).

**Figure SM2.** Differential Scanning Calorimetry (DSC) curve of the dried and ground inner bark of *Handroanthus impetiginosus*, recorded from 20 to 300 ºC at 10 ºC/min under N_2_ (30 mL/min).

**Figure SM3.** Pyrogallol calibration curve used for the determination of total polyphenol content by the Folin-Denis method. The regression equation and coefficient of determination (R²) are shown in the graph. Data represent the mean of three technical replicates measured at 750 nm. Error bars represent the standard deviation (SD).

**Figure SM4.** Concentration–response curve for 2,2-diphenyl-1-picrylhydrazyl (DPPH) radical scavenging activity of the *Handroanthus impetiginosus* lyophilized extract. The IC_50-DPPH_ was determined by nonlinear regression using a four-parameter logistic model (log[inhibitor] vs. response, variable slope). The _IC50-DPPH_ value, coefficient of determination (R²), and 95% confidence interval (95% CI) are presented in the graph. Data represent the mean of three technical replicates measured at 517 nm. Error bars represent the standard deviation (SD).

**Figure SM5.** HPLC-DAD chromatogram of the lapachol standard solution (200 μg/mL) recorded at 254 nm. The inset shows the DAD spectrum of the peak at retention time of 30.909 min, with absorption maxima at approximately 252, 277, and 335 nm.

**Figure SM6.** HPLC-DAD chromatogram of the α-lapachone standard solution (200 μg/mL) recorded at 254 nm. The inset shows the DAD spectrum of the peak at retention time of 20.872 min, with absorption maxima at approximately 252, 282, 336, and 485 nm.

**Figure SM7.** HPLC-DAD chromatogram of the β-lapachone standard solution (200 μg/mL) recorded at 254 nm. The inset shows the DAD spectrum of the peak at retention time of 29.644 min, with absorption maxima at approximately 257, 285, 339, and 441 nm.

**Table SM1.** Mean differences and adjusted *p*-values obtained by Tukey's multiple comparisons test for the cytotoxicity assays performed in HUVEC, MDA-MB-231, A549, and HepG2 cells.

| Comparison | HUVEC | | MDA-MB-231 | | A549 | | HepG2 | |
| --- | --- | --- | --- | --- | --- | --- | --- | --- |
|  | Mean difference | Adjusted  *p*-value | Mean difference | Adjusted  *p*-value | Mean difference | Adjusted  *p*-value | Mean difference | Adjusted  *p*-value |
| NC vs SC | 0.87 | >0.9999 | 0.38 | >0.9999 | -3.16 | 0.9970 | 2.24 | 0.9964 |
| NC vs PC | 14.20 | <0.0001 | 10.76 | <0.0001 | 5.70 | 0.6375 | 53.26 | <0.0001 |
| NC vs E_1_ | 43.55 | <0.0001 | 56.12 | <0.0001 | 74.14 | <0.0001 | 89.41 | <0.0001 |
| NC vs E_2_ | 12.86 | <0.0001 | 13.23 | <0.0001 | 51.93 | <0.0001 | 68.99 | <0.0001 |
| NC vs E_3_ | 1.443 | 0.9996 | 3.84 | 0.1084 | 40.57 | <0.0001 | 20.21 | <0.0001 |
| NC vs E_4_ | 2.38 | 0.9785 | 3.57 | 0.1647 | -2.97 | 0.9955 | -2.72 | 0.9843 |
| NC vs VA_1_ | 0.29 | >0.9999 | -0.93 | 0.9997 | -3.81 | 0.9877 | -4.99 | 0.7516 |
| NC vs VA_2_ | -1.18 | >0.9999 | -2.84 | 0.4290 | -3.79 | 0.9881 | -5.26 | 0.6944 |
| NC vs VA_3_ | -1.39 | 0.9997 | 1.03 | 0.9979 | -2.61 | 0.9994 | -2.74 | 0.9927 |
| NC vs VA_4_ | -0.44 | >0.9999 | 0.12 | >0.9999 | -3.41 | 0.9945 | -10.59 | 0.0323 |
| SC vs E_1_ | 42.68 | <0.0001 | 55.74 | <0.0001 | 77.29 | <0.0001 | 87.17 | <0.0001 |
| SC vs VA_1_ | -0.58 | >0.9999 | -1.32 | 0.9942 | -0.652 | >0.9999 | -7.22 | 0.2952 |
| E_1_ vs VA_1_ | -43.26 | <0.0001 | -57.06 | <0.0001 | -77.94 | <0.0001 | -94.39 | <0.0001 |
| E_2_ vs VA_2_ | -14.04 | <0.0001 | -16.07 | <0.0001 | -55.72 | <0.0001 | -74.25 | <0.0001 |
| E_3_ vs VA_3_ | -2.833 | 0.9348 | -2.80 | 0.4447 | -43.18 | <0.0001 | -22.95 | <0.0001 |
| E_4_ vs VA_4_ | -2.822 | 0.9363 | -3.45 | 0.1961 | -0.438 | >0.9999 | -7.86 | 0.2039 |
| Adjusted *p*-values < 0.05 indicate statistically significant differences. | | | | | | | | |
